# Prevalence and correlates of substance use among transgender adults: a systematic narrative review

**DOI:** 10.1192/bjo.2021.655

**Published:** 2021-06-18

**Authors:** Dean Connolly, Gail Gilchrist

**Affiliations:** 1 IoPNN King's College London; 2 IoPPN King's College London

## Abstract

**Aims:**

To understand the prevalence, patterns and correlates of substance use among transgender adults.

**Background:**

Minority stress theories suggest that the increasing rates of discrimination experienced by transgender people are precipitants of substance use. This is likely exacerbated by an inadequate provision of trans-inclusive substance misuse services. However, the exclusion of transgender people from the general substance misuse literature makes it difficult to determine the extent to which gender minority status influences substance use. A systematic review was undertaken to better understand the prevalence, patterns and correlates of substance use among this group.

**Method:**

In accordance with the PRISMA guidance, a literature search was conducted to 29th May 2019 on PubMed, PsycINFO, EMBASE and Global Health databases. Primary quantitative studies, published in the English language, that reported the prevalence, patterns or correlates/risk factors of substance use by trans people were included, with no restriction on methodological design.

**Result:**

651 unique records were identified by the search and 40 studies were included in the synthesis. While there was some suggestion of excess risk of substance use among trans people, there was insufficient evidence to estimate prevalence or quantify the risk of substance use among transgender people, relative to a cisgender population. However, this review identified several gender minority related correlates of substance use which are of relevance to clinicians working with transgender patients, including transphobic discrimination or violence, unemployment and sex work, gender dysphoria, high visual gender non-conformity and intersectional sexual minority status.

**Conclusion:**

There are currently significant gaps in the trans substance use literature, relating to the disproportionate investigation of transgender women with multiple intersectional disadvantages, who are not representative of the wider trans community. However, there is sufficient evidence to recommend screening for substance use when individuals report high levels of gender minority stress and to consider the use of integrated trauma-informed psychosocial interventions when managing problematic substance use in the transgender adult.

